# A Review of Studies Examining Stated Preferences for Cancer Screening

**Published:** 2006-06-15

**Authors:** Kathryn A Phillips, Stephanie Van Bebber, Judith Walsh, Deborah Marshall, Thabane Lehana

**Affiliations:** University of California, San Francisco; University of California, San Francisco, San Francisco, Calif; University of California, San Francisco, San Francisco, Calif; McMaster University and St. Joseph’s Hospital, Hamilton, Ontario, Canada; McMaster University, Hamilton, Ontario, Canada

## Abstract

**Introduction:**

Stated preference studies for cancer screening programs are used to understand how the programs can be improved to maximize usage. Our objectives were to conduct a systematic review of stated preference studies for cancer screening, identify gaps in the literature, and determine which types of research should be conducted in the future.

**Methods:**

We considered all studies in the PubMed database through May 2005 that measured utility-based stated preferences for cancer screening using contingent valuation or conjoint analysis. We abstracted data on 1) study characteristics and 2) study results and policy implications.

**Results:**

We found eight (of 84 identified) preference studies for cancer screening. The most commonly studied cancer was breast cancer, and the most commonly used method was contingent valuation. We found no studies for prostate cancer or physician preferences. Studies demonstrated that although individuals are able to state their preferences for cancer screening, they do not weigh test benefits and harms, and a significant percentage would choose to have no screening at all. Several studies found that test accuracy and reduction in mortality risk were important for determining preferences.

**Conclusion:**

Few studies of cancer screening preferences exist. The available studies examine only a few types of cancer and do not explore practice and policy implications in depth. The results of this review will be useful in identifying the focus of future research, identifying which screening methods may be more preferred to increase use of the programs, and developing interventions and policies that could facilitate informed and shared decision making for screening.

## Introduction

In the United States, several types of cancer screening have been recommended, and increasing the number of individuals who receive recommended cancer screenings is a health priority. Therefore, it is important to understand individual screening preferences and how the preferences can be used to develop future programs and policies. The objectives of this study were as follows:

1. Conduct a systematic review of the PubMed database for stated preference studies on cancer screening by using contingent valuation or conjoint analysis approaches to identify the numbers and types of published studies
2. Identify gaps in the literature and assess which types of research should be conducted in the future to better assess the influence of individual preferences on cancer screening decisions, clinical practice, and health policy.

Previous studies have found that patient preferences for health care interventions can have a large impact on their willingness to use services and on the resulting outcomes (1). Understanding preferences is also important because of the increasing emphasis on involving patients in decisions about their care. However, measurement, or *valuation*, of individuals' preferences for health care interventions such as cancer screening programs is a significant challenge for health care researchers because this type of information is typically obtained from surveys. The most commonly used approaches to valuation are attitude surveys, which ask respondents to rate their opinion about individual health care services, and utility-based preference surveys, which use more complex approaches that are based on economic theory. Although the terms *attitudes* and *preferences* are occasionally used interchangeably, the term *preferences* in this article refers to preferences based on economic theory — patients have preferences for health care, and they seek to maximize usefulness within the constraints of a budget. Understanding preferences, rather than simply attitudes, is particularly important for understanding the use of cancer screening because preference studies provide insights into how individuals weigh harms and benefits of tests and quantify preferences into dollars.

This review focuses on *stated preference studies — *or studies of cancer screening preferences that were measured using a theoretically based, economic approach and that used contingent valuation or conjoint analysis approaches. To our knowledge, no review of these types of preference studies of cancer screening has been done. The results will be useful in identifying areas for future research, identifying which screening methods may be preferred to increase usage, and developing interventions and policies that could facilitate informed and shared decision making about screening.

## Methods

### Definition of preference study

Several approaches are available for measuring preferences, and much confusion in the literature exists about the terms used to describe different types of studies. In this study, we limited our analyses to utility-based preference studies using contingent valuation surveys (also called *willingness-to-pay surveys*) or conjoint analysis surveys (also called *choice format stated preferences* or *discrete choice experiments*). Thus, we did not include attitude studies or preference studies used to develop health-state utility weights for use in quality-adjusted life years or other utility approaches. Contingent valuation and conjoint analysis approaches are often the most relevant to cancer screening and thus are the focus of our study. These methods measure the value of screening programs and the process of care as well as outcomes, not just health states. Furthermore, the use of these methods is increasing because of their strengths in realistically measuring choices and the harms and benefits of screening.

Contingent valuation studies use questionnaires to estimate the willingness of respondents to pay for projects or programs, typically public programs for which there is no defined market. For example, a contingent valuation of preferences for colorectal cancer screening would describe a possible screening program and ask individuals how much they would be willing to pay for such a program. Conjoint analysis studies involve comparing hypothetical scenarios by ranking, rating, or choosing scenarios. For example, respondents may be asked to choose from test A and test B after each test is described by using a combination of attributes. A conjoint analysis of preferences for colorectal cancer screening might describe different testing methods in terms of process, accuracy, and cost. Examples of each type of survey are included in Appendices A and B.

### Inclusion and exclusion criteria

We included all studies that measured stated patient preferences for cancer screening using conjoint analysis or contingent valuation. Studies that used simple rating scales to measure attitudes or studies that measured utility weights using time trade-off, standard gamble, or rating scales were excluded. A study was considered to be a cancer screening study if it examined a cancer screening program, test, or method. Studies that focused primarily on methodology of preference measurement, examined preferences for treatment, or focused on diagnosing disease characteristics (such as screening known cancers for genetic mutations) were excluded.

### Data source and search strategy

We searched the PubMed (Medline) database for studies that measured patient preferences for cancer screening, using several search strategies to identify all potentially relevant studies. Our search included all English articles through May 2005 with no limitations on start date. To identify search terms, we first reviewed the index for several known studies of quantitative preference measurement (1-4). Preliminary search results suggested that study indexing is not standardized, possibly because neither terms for preference measurement (e.g., *conjoint analysis*) nor the term *preference* are associated with a unique medical subject heading (MeSH) term in PubMed. Therefore, we combined MeSHs for the four key components of interest. The search strategy combined the following terms by using Boolean operators —* OR* within the four categories and *AND* across the categories:

1. **Cancer terms**. We used the MeSH term *neoplasm* OR the keyword *cancer*.
2. **Screening terms**. We used the MeSH terms m*ass screening* OR *mass screening/economics* and keywords *cancer* OR *screening*.
3. **Preference terms**. No MeSH term for *preferences* exists. Thus, we used a combination of the MeSH terms *patient satisfaction/economics* OR *patient satisfaction/statistics & numerical data* OR *consumer satisfaction/economics* OR *consumer satisfaction/statistics & numerical data* OR *health knowledge, attitudes, practice* as well as the keywords *preference(s)* OR *attitudes*.
4. **Quantitative methods**. We used one MeSH term, *choice behavior,* and keywords *conjoint analysis* OR *contingent valuation* OR *stated preference* OR *discrete choice* OR *willingness to pay* to capture the quantitative methods used to measure preferences.

### Study selection and coding

Two authors (SVB and KAP)Two authors (SVB and KAP) independently reviewed titles and abstracts, and all potential articles were obtained for additional review. The two authors also conducted all data abstraction and reached consensus through discussion about any disagreement. We abstracted two types of information: 1) study descriptions (cancer site, method used, objectives, and population), and 2) study results and policy implications.

## Results

We found eight stated preference studies for cancer screening (of 84 identified) (Figure). Three fourths of the studies were excluded based on the abstract or title either because they were not stated preference studies (n = 24) or because they were not relevant to cancer screening (n = 37). Fifteen of the remaining 23 citations were subsequently excluded after a full review: six were not stated preference studies and one was not relevant to cancer screening. We also excluded eight studies, even though they were cancer screening preference studies, because they focused on methodological issues.

**Figure:**
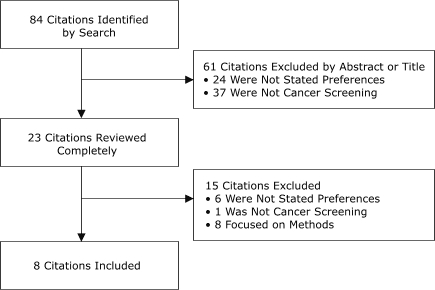
Flow chart of PubMed database search strategy and results.

Table 1 describes the included studies (4-11). The most commonly studied types of cancer were breast cancer (n = 4) and colorectal cancer (n = 3). We found no preference studies for prostate cancer screening, even though prostate cancer is the most common cancer among men, and preferences are particularly important because no consensus exists on the appropriateness of prostate cancer screening. The most commonly used method to assess preference was contingent valuation to determine willingness to pay (n = 5). Most studies administered surveys to a general population (n = 6).

Several policy implications emerged from the study results (Table 2). Studies demonstrated that individuals were able to provide meaningful responses and state their preferences for cancer screening, although many respondents did not consider the potential harms of a particular screening test, and a significant percentage would not choose any type of screening (4,8). Several studies found that test accuracy and a reduction in mortality risk were important for determining preferences (4,7,9). Researchers generally noted that preference studies could provide useful information for clinicians and policymakers in determining the net benefits of screening programs and which screening types may be most preferred, but none of the studies explored clinical or policy implications in detail.

Several studies showed that test accuracy plays an important role in preference for screening (4,7,9). Furthermore, our colorectal cancer screening studies have found that sensitivity (i.e., the ability of the test to identify those with cancer) is more highly valued than specificity (i.e., the ability of the test to correctly identify those without cancer) (12). The study by Salkeld et al (8) highlights another important finding: many people may prefer no screening at all, thus the currently available methods of screening may actually provide disutility to some individuals (i.e., they may think the tests cause more harm than good).

## Discussion

Only a few preference studies for cancer screening have been published; they assess few types of cancer and have a limited range of questions. We were surprised that we did not identify any studies of physicians' predictions about their patients' preferences. Physician recommendations for screening have consistently been found to be strong predictors of screening usage, so it is important to understand how well physicians' views of preferences actually reflect patients' preferences. We found in our ongoing study of colorectal cancer screening that physicians' views of patients' preferences were not congruent with what patients reported they preferred (12). In particular, physicians were much more likely to predict that patients would prefer no screening when the patients actually preferred screening, which may explain why some physicians do not always offer screening to their patients.

Our review suggests that although preference studies can provide useful findings that can improve our understanding of cancer screening, more research needs to be completed. We found that test accuracy plays a role in screening preference. The preference for sensitivity over specificity suggests that individuals tend to prefer false-positive results over false-negative results. This may help explain the willingness of individuals to receive complete-body computed tomography scans and for women aged 40 to 50 years to undergo mammography screenings, despite the high rate of false-positives for such procedures. Schiffner et al (9) confirm that patients may not understand the potential drawbacks of having false-positive results. This finding by Salkeld et al (8) that many people prefer no screening is important from a methodological perspective because it suggests that preference surveys should consider measuring not only preferences for screening but also preferences for no screening.

Our findings also suggest that it may be necessary to develop new methods of screening that better address patient preferences. Although we recognize the ongoing debate about whether the goal of preventive public health programs is to maximize participation in screening or maximize the usefulness of the screening, our study did not address these issues. Regardless, preference information can be useful for meeting either goal.

None of the studies explored in detail the implications of their results for clinical practice and health policy. This research gap is surprising given the current emphasis on more patient participation and shared, or informed, decision making between patients and providers. For example, some guidelines for mammography screening for women aged 40 to 50 years recommend that screening decisions be based on preferences and shared decision making. In general, research on informed decision making and decision aids is based on attitude data rather than true preference data, although preference data could provide additional insight into what patients want and how to elicit preferences for use in decision making. Thus, one key area for future research is the development of mechanisms that enable the use of preference data. During the development of such mechanisms, researchers will need to consider how to simplify the often time-consuming surveys used to measure preferences and develop efficient ways to use preference data in clinical practice. Similarly, preferences need to be incorporated into health policies. For example, our finding that many people would prefer colonoscopy over other forms of colorectal cancer screening provides important information, because many private insurers and government health systems do not currently provide coverage for colonoscopies (13).

Our study has limitations. Our literature search may not have identified potentially relevant studies that have not been indexed in PubMed or published in English. In addition, because the studies we found had varying methods (e.g., contingent valuation, conjoint analysis), research questions, and cancer types, we were unable to conduct a quantitative analysis such as a meta-analysis. We excluded other types of utility studies because they were not as relevant to this review, but future researchers could examine these studies.

Although we identified only a few studies of cancer screening preferences, the published studies have provided some useful results. More research is needed to identify preferences to help clinicians and decision makers improve screening programs.

## Figures and Tables

**Table 1:** Reviewed Studies for Stated Preferences for Cancer Screening (N = 8)

**Study**	**Cancer Type**	**Preference Method**	**Objective**	**Population and Sample Size**
Frew et al (5)	Colorectal	Contingent valuation	To examine willingness to pay for fecal occult blood testing and flexible sigmoidoscopy	General population (N = 2000)
Gyrd-Hansen (6)	Breast	Conjoint analysis	To assess all costs incurred by and effects of introducing mammography screening	General population (N = 207)
Gyrd-Hansen and Sogaard (4)	Breast, colorectal	Conjoint analysis	To determine population preferences for cancer screening programs	General population (N = 750)
Liang et al (7)	Breast	Contingent valuation	To assess acceptability of a new noninvasive breast cancer diagnostic test intended to triage women in need of biopsy	Patients (N = 43)
Salkeld et al (8)	Colorectal	Conjoint analysis, contingent valuation	To elicit preferences for colorectal cancer screening by fecal occult blood testing	General population (N = 301)
Schiffner et al (9)	Skin	Contingent valuation	To determine the difference between patients' confidence (as measured by willingness to pay) in current diagnostic methods and a diagnostic method promising 100% accuracy	Patients (N = 210)
Wagner et al (10)	Breast	Contingent valuation	To examine willingness to pay for mammography among five ethnic groups	General population (N = 1465)
Wordsworth et al (11)	Cervical	Contingent valuation	To assess the value of cervical smear (Papanicolaou) testing	General population (N = 2000)

**Table 2:** Summary of Primary Results and Policy Implications of Reviewed Studies for Stated Preferences for Cancer Screening (N = 8)

**Study**	**Primary Results**	**Policy Implications**
Frew et al (5)	Willingness to pay for flexible sigmoidoscopy was similar to likely resource costs of screening for sigmoidoscopy and fecal occult blood testing.	The study helps establish extent to which a new technology would be valued by the public.
Gyrd-Hansen (6)	The study involved a cost-benefit analysis using preference data and found that net benefits are maximized when mammography screening is targeted biennially to women aged 50-74 years.	Preference studies can be used to identify inferior programs.
Gyrd-Hansen and Sogaard (4)	Preferences for colorectal and breast cancer screening were primarily explained by positive utility associated with reducing mortality risk and disutility from out-of-pocket expenses.	It is important to identify the relative importance of program attributes to identify and exclude programs that consume more resources and provide less utility.
Liang et al (7)	Women would find noninvasive triage tests for breast cancer acceptable or preferable to biopsy if they were equally accurate.	New technologies should focus on decreasing discomfort as well as increasing test accuracy.
Salkeld et al (8)	Three characteristics of colorectal cancer screening varied: benefits (deaths prevented), harms (unnecessary colonoscopy), notification policy (test result). 12% always preferred no screening. 32% would always choose the screening method that provides the most survival benefits (i.e., harms of screening were irrelevant).	In any future national screening program, careful consideration should be given to selection of screening tests based on the community's assessment of benefits, harms, costs, and other characteristics.
Schiffner et al (9)	Patients underestimated the actual test accuracy for malignant melanoma. A distinct gap was found between patients' level of confidence in current methods and a hypothetical tool with 100% safety.	Accuracy is highly valued but not well understood by patients. Better information about diagnostic accuracy is necessary to increase patients' knowledge of and confidence in tests.
Wagner et al (10)	Willingness to pay differed by race and ethnicity.	Preference studies that do not account for ethnic differences may be overstating net benefits to society.
Wordsworth et al (11)	The value women place on having a Papanicolaou test is more than the test's actual costs to UK National Health Service for providing the service.	Willingness-to-pay information can be useful for policy makers.

## Appendices

### Appendix A. Conjoint Analysis Example

(Adapted from Salkeld et al [8].)

Interviewees were provided with the following information about colorectal cancer screening:

- They were given background information on the screening (e.g., diagnosis in absence of screening, information about fecal occult blood testing, opportunity costs of a screening program).
- They were asked to imagine that they had been invited by their local doctor (general practitioner) to have this test for free.
- They were given a description of two identical colorectal cancer biennial screening programs for a population of 10,000 men and women aged 50 to 69 years.
- They were told that the programs were identical except for three characteristics for which standardized descriptions were provided: benefits (number of colorectal cancer deaths prevented), harms (number of colonoscopies resulting from a false-positive fecal occult blood test result), and notification policy (whether they would be notified of a negative test result).
- They were given a description of the colonoscopy procedure and a statement on the rate of complications arising from colonoscopies performed on the screened population.

Interviewees were then given a scenario and a question, such as: "Could you please compare the two programs, and tell me whether you would prefer Program A or Program B or whether you would prefer not to have the screening test?" An example follows:

**Table:** Sample Scenario (per 10,000 Men and Women Screened Over 10 Years)

**Characteristic**	**Program A**	**Program B**
Number of colorectal cancer deaths prevented	8	14
Number of unnecessary colonoscopies resulting from false-negative tests	2400	8400
Notification of negative test result	Yes	Yes

Question: Which would you prefer? Program A, Program B, or no screening?

### Appendix B. Willingness-to-Pay Example

(From Corso et al [14])

"I'd like to ask you a few questions about food safety."

(Interviewee is given one of two scenarios.)

**Prevention scenario:** "Imagine that you are planning a trip to a foreign country where for every 100,000 people visiting, 400 people contract a virus from eating contaminated food. If you get the virus, the only symptom is a slight yellowing of the skin for 2 or 3 days. The virus causes no other discomfort and does not interfere with any of your activities. However, studies have shown that for every 100 people who get the virus, one will die. Further, there is no treatment at this time. Fortunately, there is a U.S. medication available that will protect you from getting the virus in the first place, no matter what foods you eat while traveling. This medication has NO side effects. Tests have shown that this preventive medicine will decrease your risk of getting the virus by 50%. Thus, your overall chances of dying from this illness can be reduced from 4 in 100,000 to 2 in 100,000 if you take the preventive medicine."

**Treatment scenario: **"Imagine that while traveling in a foreign country you notice that your skin has been slightly yellow for 2 or 3 days. While you experience no other discomfort and have been able to conduct your normal activities, you decide to visit a local clinic run by U.S. doctors. The doctors tell you that you have contracted a virus, probably from eating contaminated food, where for every 100,000 people who have the virus, 4 will die. Fortunately there is a U.S. medication available at the clinic. This medication has NO side effects. Tests have shown that this medication will reduce your chance of dying by 50%. Thus your overall chance of dying from this virus can be reduced from 4 in 100,000 to 2 in 100,000."

(Interviewee is then asked the following:)

"Would you consider taking this medication?"

**<If NO>** "What is the main reason you would not be willing to take the medication?"

**<If YES>** "Now assume you would have to pay some money to get this medication — insurance would not cover it. Considering your current income and other household expenses, would you pay:<$50, $100, $200, $400>for this medication?"

**<If YES>** "Would you buy this medication if the out-of-pocket cost was:<$100, $200, $400, $800>?"

**<If NO>** "Would you buy this medication if the out-of-pocket cost was:<$20, $50, $100, $200>?"
